# DiGeorge Syndrome Complicated by Secondary Antiphospholipid Syndrome Presenting With Vascular Thrombosis

**DOI:** 10.1002/ccr3.71907

**Published:** 2026-01-20

**Authors:** Aziz‐ur‐Rahman Khalid, Islam Rajab, Sakeena Saife, Anwar Zahran, Ghassan Makhoul, Hasan Munshi, Ibraheem Chaudhry, Robert Lahita

**Affiliations:** ^1^ St. Joseph University Medical Center Paterson New Jersey USA; ^2^ Department of Medicine, Faculty of Medicine and Health Sciences An‐Najah National University Nablus Palestine; ^3^ Institute for Autoimmune and Rheumatic Disease, St. Joseph Health Wayne New Jersey USA

**Keywords:** 22q11.2 deletion, antiphospholipid syndrome, DiGeorge syndrome, thrombosis, vascular complications

## Abstract

DiGeorge syndrome (22q11.2 deletion syndrome) is a congenital disorder typically identified in infancy, but adult presentations may feature autoimmune and thrombotic complications. We report a 30‐year‐old woman with known DiGeorge syndrome who presented with progressive right lower extremity pain. She had a recent history of transverse sinus stenting and was on dual anti‐platelet therapy with anticoagulation. Evaluation revealed left internal jugular vein thrombosis, abnormal distal pulses, and positive antiphospholipid antibodies. Her symptoms were attributed to vascular compromise in the setting of autoimmune thrombosis. This case highlights the need for vigilance for late‐onset thrombotic manifestations in adults with DiGeorge syndrome, especially following invasive procedures, and reinforces the value of multidisciplinary and immunologic assessment in complex cases.

## Key Clinical Message

Neuropathic pain in adult DiGeorge syndrome may signal underlying autoimmune vascular pathology such as antiphospholipid syndrome. This rare presentation underscores the need for heightened clinical suspicion and multidisciplinary evaluation in post‐procedural patients, where early recognition of thrombosis can prevent irreversible ischemic damage and guide immunologic and anticoagulant therapy.

## 1 Introduction

DiGeorge syndrome (22q11.2 deletion) is a multi‐system disorder involving congenital heart disease, immune dysfunction, and endocrine abnormalities [1]. While often diagnosed in infancy, adult survivors may later present with autoimmune or thrombotic complications.

While the classic triad includes cardiac anomalies, immunodeficiency, and hypocalcemia, adult patients may experience complications not traditionally associated with the syndrome, such as vascular thrombosis and autoimmune coagulopathies. Autoimmune manifestations have been reported in about 20%–30% of adults with the 22q11.2 deletion syndrome, including cytopenias, autoimmune endocrinopathies, and systemic autoimmune diseases. Immune dysregulation, defective central tolerance, and chronic inflammation might predispose to endothelial dysfunction and a prothrombotic state. Indeed, no specific deletion breakpoint has been convincingly related to thrombosis so far, but autoimmune complications are relatively more common in partial DiGeorge phenotypes displaying residual thymic function [2, 3].

We report a rare case of DiGeorge syndrome complicated by antiphospholipid syndrome presenting with thrombosis and neuropathic symptoms.

## 2 Case Presentation (History/Examination)

A 30‐year‐old woman with DiGeorge syndrome presented with progressive right toe discoloration and 3 days of worsening pain, following 2 months of neuropathic leg symptoms. Two months earlier, she underwent transverse sinus stenting and was maintained on dual anti‐platelet therapy and apixaban for prior thrombosis.

The patient described the discoloration of her toes as progressively worsening, accompanied by cold sensation and increasing difficulty with ambulation. She denied fever, trauma, or prior episodes of peripheral ischemia. She had a history of learning difficulties and mild dysmorphia but was functionally independent.

She was afebrile with bluish discoloration and reduced distal pulses in the right foot. Allodynia was present on the dorsal foot. Left jugular tenderness was noted. Cardiac and pulmonary exams were unremarkable.

## 3 Differential Diagnosis, Investigations, and Treatment

**FIGURE 1:**
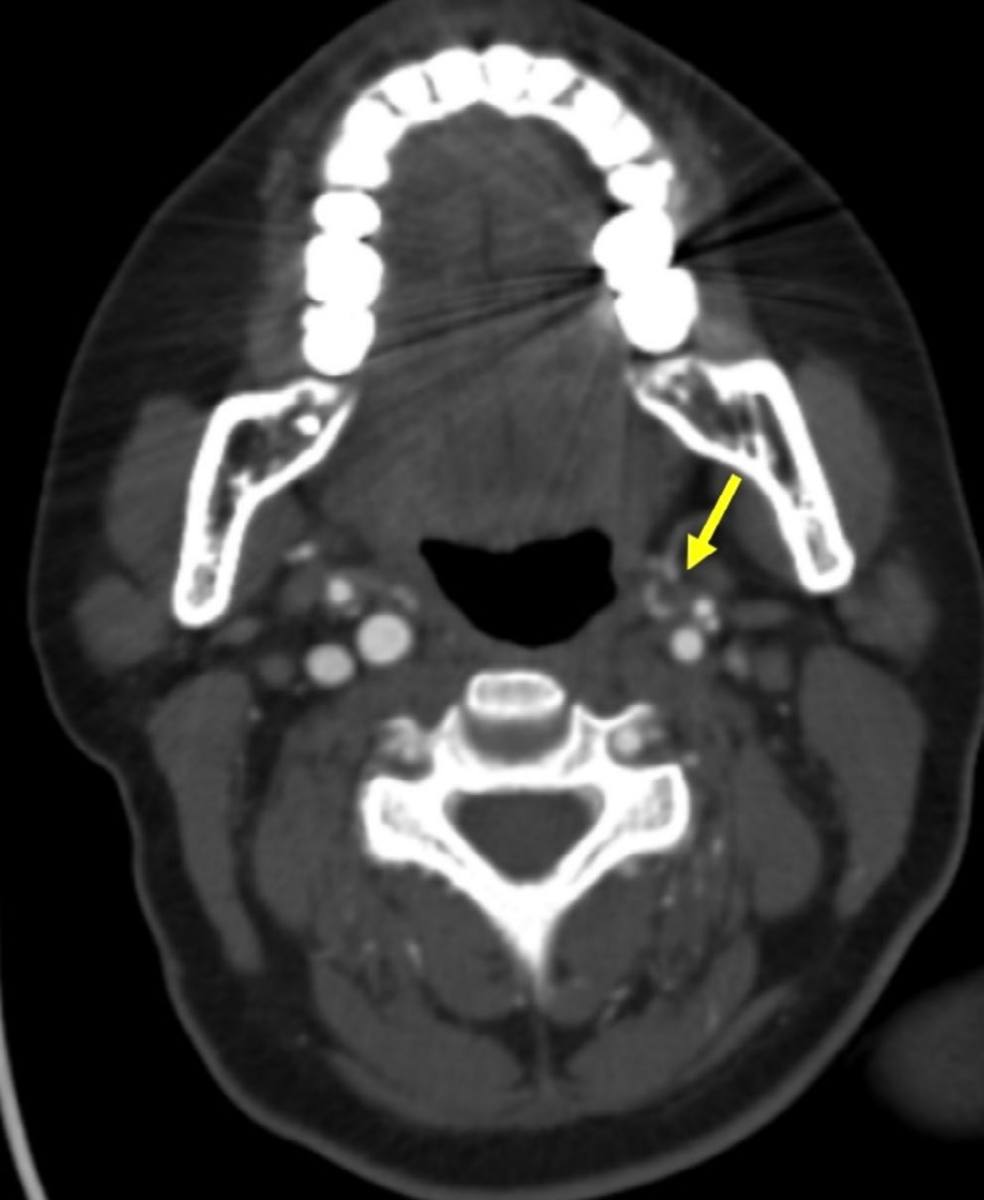
CT angiogram of the head shows nonopacification of the left proximal internal jugular vein (arrow), consistent with thrombotic occlusion.

Brain MRI showed no infarct or hemorrhage. CTA of the head revealed a nonopacified left internal jugular vein consistent with thrombotic occlusion (Figure 1). Laboratory evaluation showed normal inflammatory markers and serum calcium levels. Laboratory tests showed normal levels of inflammatory markers, such as C‐reactive protein of 2.1 mg/L (ref < 5) and erythrocyte sedimentation rate of 14 mm/h (ref: 0–20 mm/h). Serum calcium levels were normal at 9.2 mg/dL (ref: 8.6–10.2 mg/dL). Platelet count was 212 × 10^9^/L (ref: 150–400 × 10^9^/L). Coagulation profiles were positive for lupus anticoagulant, positive for anticardiolipin IgG antibodies of 58 GPL units (ref < 20 GPL units), positive for anti‐β2‐glycoprotein I IgG antibodies of 46 units (ref < 20 units), meeting laboratory criteria for antiphospholipid antibodies. no evidence of active systemic lupus erythematosus. the patient's anticoagulation was switched from apixaban to warfarin, targeting an INR of 2–3, in recognition of her high‐risk antiphospholipid syndrome and history of arterial thrombosis. Dual antiplatelet therapy with aspirin and clopidogrel was continued for a total of 3 months post‐stenting, after which it was de‐escalated to single antiplatelet therapy with aspirin alone. Platelet counts, bleeding parameters, INR, and clinical signs of recurrent thrombosis were closely monitored during hospitalization and follow‐up, ensuring safe and effective management of both stent patency and thrombotic risk. During follow‐up, the patient showed improvement of the dischromia of the toes and neuropathic pain. There were no new thrombotic or bleeding events. She is currently being maintained on anticoagulation therapy with careful hematology and neurology follow‐ups.

## 4 Conclusion and Results

This case highlights adult‐onset thrombosis and neuropathic pain in DiGeorge syndrome with secondary APS. The coexistence of antiphospholipid antibodies in a patient with 22q11.2 deletion underscores the potential for autoimmune‐mediated vascular complications in adult survivors of congenital syndromes. As more patients with partial DiGeorge phenotypes reach adulthood, clinicians must remain vigilant for late‐onset complications, including autoimmunity and thrombosis. A high index of suspicion, prompt imaging, and multidisciplinary coordination are critical to achieving favorable outcomes in such complex presentations.

## 5 Discussion

DiGeorge syndrome results from a 22q11.2 micro‐deletion affecting derivatives of the third and fourth pharyngeal pouches. Many individuals also exhibit characteristic facial features, developmental delays, and neuropsychiatric disorders [4, 5].

Autoimmune complications affect up to 30% of adults with DiGeorge syndrome. Our patient's antiphospholipid antibodies and thrombosis point to secondary antiphospholipid syndrome (APS) as a key contributor [6, 7].

In this patient, the presence of left internal jugular vein thrombosis in association with persistently positive antiphospholipid antibodies provides strong support for the diagnosis of secondary antiphospholipid syndrome. The hallmark features of APS include arterial or venous thrombosis occurring in the setting of pathogenic autoantibodies to phospholipid‐binding proteins. In spite of the fact that APS has been rarely described in association with DiGeorge syndrome, one of the features of 22q11.2 deletion may be immune dysregulation, which can promote the production of autoantibodies and endothelial injury, thereby predisposing individuals to thrombosis [8].

Recent stenting and immobility likely contributed to thrombotic risk, which introduced vascular manipulation and prolonged immobility, both risk factors for thromboembolism. Additionally, neuropathic pain and motor deficits in the right lower limb may have been exacerbated by microvascular ischemia or post‐procedural nerve injury, though definitive localization was limited by symptom overlap [9].

Dual antiplatelet therapy was concurrently maintained, despite the sinus stenting procedure, as a temporary measure to lower the risk of stent thrombosis. Additionally, with potential platelet dysfunction in both antiphospholipid syndrome and DiGeorge syndrome, risk/benefit analysis was evaluated. Platelet count and bleeding parameters were cautiously followed for observation in both hospitalized patients and follow‐up assessments for the desired course of dual anti‐platelet therapy [10].

This case underscores the importance of considering adult‐onset autoimmune and thrombotic complications in patients with DiGeorge syndrome. The presence of recurrent thrombosis and vascular symptoms should prompt a comprehensive immunologic and hematologic evaluation, including testing for antiphospholipid antibodies, especially in patients undergoing surgical or interventional procedures [11].

Management of such patients requires a multidisciplinary approach. Anticoagulation remains the mainstay of therapy in APS‐related thrombosis, though duration and intensity must be tailored to the patient's thrombotic history and bleeding risk. In patients with known congenital syndromes and evolving vascular complications, close coordination between neurology, hematology, immunology, and vascular medicine is essential [8].

Table 1 summarizes previously reported adult cases of DiGeorge syndrome complicated by autoimmune or thrombotic events, adapted from published case reports (Gu et al. [12]; Damlaj et al. [13]; Mkaouer et al. [6]; Sun et al. [14]).

**TABLE 1:** Published cases of adult DiGeorge syndrome with autoimmune or thrombotic complications.

Author (Year)	Age	Gender	Primary complication	Immunologic findings	Diagnosis	Treatment	Outcome
Gu et al. (2023) [12]	18	M	Refractory cytopenias, ALPS‐like	↑ DNT cells, ↓ naïve T cells, 22q11.2 del	Partial DiGeorge + ALPS‐like	Sirolimus	Remission, ↓ lymph nodes
Damlaj et al. (2013) [13]	18	F	Severe AIHA	+Coombs, prior ITP	DiGeorge + AIHA	Steroids, IVIG, rituximab, PLEX, splenectomy	Full remission post‐PEX
Mkaouer et al. (2024) [6]	21	F	Autoimmune diabetes, thyroiditis	+GAD Abs, +Anti‐TPO	DiGeorge + Polyglandular Autoimmunity	Insulin, thyroid hormones	Stable glucose & thyroid
Sun et al. (2023) [14]	20	F	SLE	+ANA, +dsDNA, ↓ platelets	DiGeorge + SLE	Prednisone, HCQ, azathioprine	Symptoms resolved, ↑ platelets

Abbreviations: AIHA, Autoimmune Hemolytic Anemia; ALPS, Autoimmune Lymphoproliferative Syndrome; ANA, Antinuclear Antibodies; Anti‐dsDNA, Anti‐Double Stranded DNA Antibodies; Anti‐TPO, Anti‐Thyroid Peroxidase Antibodies; DNTs, Double‐Negative T Cells; GAD Abs, Glutamic Acid Decarboxylase Antibodies; ITP, Immune Thrombocytopenic Purpura; IVIG, Intravenous Immunoglobulin; PLEX, Plasma Exchange.

## Author Contributions

**Aziz‐ur‐Rahman Khalid:** methodology, project administration, resources, supervision, visualization, writing – review and editing. **Islam Rajab:** methodology, project administration, resources, supervision, visualization, writing – review and editing. **Sakeena Saife:** conceptualization, methodology, supervision, writing – original draft. **Anwar Zahran:** methodology, writing – review and editing. **Ghassan Makhoul:** methodology, writing – review and editing. **Hasan Munshi:** methodology, writing – review and editing. **Ibraheem Chaudhry:** methodology, writing – review and editing. **Robert Lahita:** methodology, writing – review and editing.

## Funding

The authors have nothing to report.

## Consent

As this is a case report, written consent was obtained for the purpose of this paper.

## Conflicts of Interest

The authors declare no conflicts of interest.

## Data Availability

The data that support the findings of this study are available on request from the corresponding author. The data are not publicly available due to privacy or ethical restrictions.
